# A test of four evolutionary hypotheses of pregnancy food cravings: evidence for the social bargaining model

**DOI:** 10.1098/rsos.170243

**Published:** 2017-10-18

**Authors:** Caitlyn Placek

**Affiliations:** Department of Anthropology, Ball State University, Muncie, IN, USA

**Keywords:** pregnancy cravings, South India, behavioural immune system, resource scarcity, son preference, social bargaining

## Abstract

The onset of cravings for items not typically desired is often considered a hallmark of pregnancy. Given the ubiquity of cravings, this phenomenon remains surprisingly understudied. The current study tested four hypotheses of pregnancy food cravings: behavioural immune system, nutrient seeking, resource scarcity and social bargaining. The research took place in Tamil Nadu, South India, with pregnant women residing in rural villages (*N *= 94). Methods included structured interviews and anthropometric measures. Findings revealed that unripe mango and unripe tamarind were the two most frequently mentioned food cravings among this population, but were not sufficiently supported by the *a priori* models. Results confirmed that the social bargaining model was the best explanation for the etic category of toxic/pathogenic food items, suggesting that pregnant women crave dangerous foods when experiencing heightened social pressures. Finally, toxicity/pathogenicity was a confounding factor for the nutrient seeking and resource scarcity models, calling into question the validity of these models in adverse environments. Overall, these findings present important implications for research on pregnancy food cravings, such that in resource-scarce and pathogen-dense environments, cravings might target teratogenic items that signal a need for increased social support.

## 1. Introduction

Across cultures, a craving for items not typically desired is often considered a hallmark of pregnancy. Women are known for craving sweets, fruits, calorie-dense foods, odd combinations such as ‘pickles and ice cream’, or pica substances, such as clay and chalk [1–3]. Despite the widespread occurrence of cravings, this phenomenon remains relatively understudied [1]. Evolutionary theories proposed to explain food cravings include a need to seek foods to either satisfy the energetic demands of the growing fetus, or to replenish nutrients lost from nausea, vomiting and food aversions in the first trimester [4,5]. Research has also suggested that cravings for nutritional foods could operate as a buffering strategy to store nutrients in environments that lack adequate access to resources [6]. While the biological and environmental correlates are essential to investigate, food cravings also could serve a psychosocial function. This study introduces a new hypothesis that cravings for harmful foods function as a bargaining strategy when pregnant women have increased pressure to fulfil cultural norms of motherhood. Herein these various hypotheses are tested among a sample of pregnant Tamil women from South India.

Pregnancy is unique in that several immunological shifts occur to support fetal development [7]. Researchers once thought that pregnancy was a period of total immunosuppression [8]; however, empirical evidence now shows that women are capable of a robust immune response while also withstanding a series of changes in cell-mediated immunity that aim to support implantation and placentation [7,9,10]. These changes leave the woman more vulnerable to certain pathogens and toxins, such as influenza, hepatitis E, herpes-simplex viruses and malaria [7,10,11]. At the same time, environmental and dietary toxins threaten the development of the fetus [4,12]. The woman's responses to these threats, such as nausea, vomiting, meat aversions and cravings for non-food substances (pica), have been hypothesized to serve as ‘behavioural immune responses’ that aid the mother in resisting pathogen infection and toxin ingestion [3,5,13,14]. Food cravings are considered an additional tool that assists in pathogen avoidance [5]. For instance, cravings for unripe fruits could reflect an underlying need for vitamin C, which in turn reduces teratogenic effects of environmental toxins [5,15]. Unripe fruits, such as raw mango, contain tannins, which are useful in treating ulcerated or inflamed tissues and also provide a stable source of antioxidants [16]. In addition, metabolic shifts early in pregnancy impact bioavailability of dietary antioxidants because the placenta produces high numbers of oxygen-based molecules [17]. In turn, women experience an increased need for foods rich in antioxidants. Thus, under the umbrella of the behavioural immune hypothesis of pregnancy cravings, women should desire fruits rich in antioxidants during the first trimester of gestation to serve as a mechanism of protection. Support for this hypothesis stems from cross-cultural studies among women who report cravings for fruit and fruit juices [2,18].

Cravings that follow the period of nausea and vomiting in the first trimester might function to replenish lost nutrients [2,4]. By the tenth week of pregnancy, women begin to store excess fat, reduce physical activity and consume foods high in calorie content to support fetal growth and development [4,17,19]. This hypothesis, called the ‘nutrient seeking hypothesis’, was initially proposed by Hook [4], and is supported by studies conducted in both Western and non-Western settings [19–21]. Specifically, research in Tanzania found that pregnant women who experienced food aversions in the first trimester subsequently craved a variety of foods rich in both calories and nutrients, thus compensating for nutrient loss from aversions [22]. A different, but not mutually exclusive, hypothesis claims that pregnancy cravings targeted toward nutrient-dense foods, and not simply foods high in calories, could function as a buffering strategy during periods of heightened resource scarcity, and not coincide with months pregnant [6]. The ‘resource scarcity hypothesis’ was coined from a study in southern Ethiopia, where pregnant women desired meat and vegetables, two items that were locally unavailable due to lack of sufficient resources [6]. Among the Turkana and Datoga women of East Africa, the majority of women reported aversions towards maize, a staple food, and craved meat and dairy, with cultural explanations centred on maintenance of maternal health and strength [23].

Alternatively, pregnancy cravings could function as a signalling strategy to gain social support among those who perceive an uncertain future for their offspring. Hagen [24] proposed a similar strategy among postpartum women who used depression to bargain for social support [24]. Prior research indicates that individuals who perceive a lack of social support engage in emotional eating [25,26]. In pregnancy, aboriginal women reported frequent bingeing and purging in response to psychological distress [27], whereas expectant mothers in Tamil Nadu showed heightened psychological distress in association with consumption of forbidden pica substances [13]. Owing to patrilineal descent and the expensive practice of dowry, Indian women often face familial and community pressure to give birth to a son [28]. This pressure is associated with symptoms of depression during the prenatal and postpartum periods [29,30], and is sometimes accompanied by abuse and denying pregnant women foods that they crave [31]. Indian women who have had multiple girl children report heightened distress over failure to produce a son and their lack of resources to provide a dowry for multiple girl children [32,33]. Cravings for items that threaten the pregnancy could, therefore, be an expression of this inability to conform to cultural norms of the ‘ideal family’. Furthermore, failure of South Indian family members to satisfy women's needs is thought to lead to cravings for items that are potentially harmful to the mother and fetus [34]. Given these lines of evidence, cravings for toxic and pathogenic items, as well as those viewed as dangerous if consumed in high quantities, such as unripe mango, unripe tamarind and papaya [13,35], are predicted to function as a social bargaining strategy among women who have higher fertility, feel pressure to have a son, heightened resource scarcity and experience psychological distress.

## 2. Study aims

This study tested the likelihood of the above hypotheses of food cravings among pregnant women in Tamil Nadu, India to determine the ‘best’ fitting model for cravings. Specifically, the following hypotheses were considered: (i) *behavioural immune system*: food cravings will be rich in vitamin C and antioxidants, they will occur during the first trimester and will be associated with nausea and vomiting; (ii) *nutrient seeking*: women will crave calorie-rich and nutritionally dense foods; these will be positively associated with months pregnant and negatively associated with anthropometric indicators of health; (iii) *resource scarcity*: food cravings for nutritious foods will be correlated with low dietary diversity and resource scarcity, but will have no relationship with months pregnant; (iv) *bargaining strategy*: psychological distress, resource scarcity, pressure to have a son and number of children will be correlated with cravings for ‘dangerous’ food items.

## 3. Study population

This study focused on a population of pregnant women located in thirteen agricultural villages in Tiruvannamalai, Tamil Nadu, India (12.23° N, 79.07° E). Pregnancy in Tamil Nadu is considered a period of increased desire (*acai*), which is the overarching cultural explanation for specific cravings for foods and non-foods (pica substances; [13]). To quell pregnancy cravings and ensure a positive pregnancy outcome, family and community members host a special ceremony, called *Vallaikappu*, where women engage in *pujas* (religious rituals) and receive gifts of food, sarees and bangles [34]. Women in this region are encouraged to avoid consumption of black foods and fruits that increase bodily heat, such as papaya, unripe mango and unripe tamarind [13,36]. Despite cultural norms that oppose consumption of heat-causing fruits, pregnancy cravings for these items are common [13,36]. Food cravings among this particular population have not been investigated in the context of evolutionary theory.

Resource scarcity is prevalent in this region. A study conducted with this population of Tamil women found that nearly half of the study participants suffered from household food insecurity [36]. In a population near Tiruvannamalai, nearly 75% of participants were highly food insecure [29]. More broadly, India is a country with heightened food insecurity where approximately 32% live below $1.00 per day [37]. Pathogen density in India is also high. The death rate due to communicable disease is at 41% [37], compared with the global average of 24.9% [38]. Pregnant Indian women experience heightened risk of contracting malaria [39], are exposed to water-borne illnesses [40] and sexually transmitted diseases, such as HIV [41]. Finally, pressures to produce a sizeable dowry for marriage have led to son preference across Indian states [42]. In Tamil Nadu, the high cost of dowry has resulted in beliefs of an ideal family consisting of one son and one daughter [43]. Women who have given birth to more than one daughter feel increased pressure to produce expensive dowries and therefore use various methods to either avoid giving birth to a third child, terminate the pregnancy, or engage in female infanticide [42,43].

## 4. Methodology

This study relied on Agar's [44] ‘ethnographic funnel’ method that starts with a general question (e.g. ‘What do women crave during pregnancy?’) and then hones in on a more specific set of questions that lead to hypothesis testing. Early stages of this project consisted of informal interviews with women in the community (*n* = 10) who told stories of common pregnancy cravings. Information gathered at this stage led to a semi-structured questionnaire that asked community women (*N* = 54) specific questions about pregnancy cravings and the consequences of consuming the mentioned items. Results for these stages are reported in [13] and [36]. In the final step of data collection, *N* = 95 pregnant women underwent structured interviews that assessed their aversions, cravings and consumption patterns in pregnancy. Women also addressed questions focusing on resource scarcity and pathogen exposure. Finally, anthropometric measurements were collected. Participants were recruited from primary health centres (PHCs) located within each village. Women in this region are given incentives for registering with PHCs, therefore, this sample is probably inclusive of all women who were pregnant in this region at the time of data collection. All women provided informed consent before beginning the structured survey. The Washington State University Institutional Review Board approved this research.

## 5. Analysis

Data were analysed in R v. 3.3.3 (3 June 2017) for Macintosh and can be found publicly on Figshare [45]. The following hypotheses were tested: behavioural immune system, nutrient seeking, resource scarcity and social bargaining. Univariate analyses and multivariate modelling were used to test the hypotheses. First, free-list data for food cravings were graphically represented to determine the most frequently craved items. Additional outcome variables were created from free-list data to represent the different model predictions. Next, models were generated to test each hypothesis individually. Since outcome measures differed for each model, statistical tests were chosen by examining the distribution of variables and appropriate tests were determined by comparing model options based on Akaike information criterion (AIC) [46]. For example, tests for negatively skewed ordinal variables were selected based on the lowest AIC score between Poisson and negative binomial regressions. Overall model strength was determined by using McFadden's *R*^2^-values for Poisson and negative binomial models, and Nagelkerke *R*^2^-values for logistic regression models. The strength of individual predictors was determined by coefficient estimates (Poisson and negative binomial models), odds ratios (logistic regression models), 95% CIs and *p*-values. Since these models are not mutually exclusive, some predictor variables are included in more than one model. Table 1 presents each model including outcome and predictor variables, and specific analytic details for each model are described below.

**Table 1.** Summary of outcome and predictor variables for each study model.

model	outcome variables	predictor variables
behavioural immune system	fruit cravings	nausea
	unripe mango cravings	vomiting
	unripe tamarind cravings	months pregnant
nutrient seeking	total calories	triceps thickness
	total cravings for nutritional foods	BMI
		months pregnant
		toxicity/pathogenicity
resource scarcity	total cravings for nutritional foods	triceps thickness
		BMI
		dietary diversity
		food insecurity
		months pregnant
social bargaining	toxicity/pathogenicity	psychological distress
	‘hot’ food cravings	food insecurity
	unripe mango cravings	pressure to have a son
	unripe tamarind cravings	number of living children
		pressure × living children

### 5.1. Behavioural immune system

The behavioural immune system predicts that pregnant women experience cravings for micronutrients that are lost due to increased nausea and vomiting that occur during the early months of pregnancy. According to this hypothesis, cravings will focus on foods rich in antioxidants, such as unripe and regular fruits [5]. To test this hypothesis, logistic regression models were fitted to predict cravings for ripe and unripe fruits. A dichotomous variable was created that measured cravings for ripe fruits, and the most frequently craved food items, unripe mango and unripe tamarind, were also transformed into dichotomous outcome variables (presence = 1, absence = 0). Predictor variables included the presence/absence of nausea and vomiting (yes = 1, no = 0), and months pregnant.

### 5.2. Nutrient seeking

This model predicts that women will crave calorically dense or nutritionally rich foods, and these will be positively associated with months pregnant, and indicators of nutritional need, such as triceps thickness and BMI. Calories per food item were computed using the Nutritive Value of Indian Foods measure by Gopalan *et al*. and is accessible as freeware (http://bit.ly/ncalculator). Foods not listed in this source were located on the South Indian foods calorie chart (http://southindianfoods.in/south_indian_food_caloriechart.html). Next, a calorie-density composite score was computed for each woman's free-listed cravings. An outcome variable for nutritious foods was created from the free-listed cravings. Foods that were considered nutritious were situated within a South Indian context of what is considered healthy to consume in pregnancy: vegetables, ripe fruits, meat, eggs, fish and dairy products [34]. Unripe fruits were not considered healthy, because they are perceived to lead to spontaneous abortion if consumed in high quantities [13], and were thus excluded. Predictor variables included months pregnant, triceps thickness and BMI. To control for toxicity and pathogenicity of each food item, a control variable was created and included in the models. To create this variable, each food was given a ‘1’ if it was potentially toxic or pathogenic (e.g. vegetables or meat), and a ‘0’ if not.

### 5.3. Resource scarcity

The resource scarcity model indicates that women should experience cravings for nutritious foods because their diets are limited to a few food categories [6]. The measure of nutritious food consumption was selected as the outcome variable. Predictor variables included total food insecurity, dietary diversity, triceps thickness and BMI. Months pregnant was included as a control measure and was predicted to have no association with the outcome. Food insecurity was measured with the six-item short-form measure of food security that has demonstrated adequate validity and reliability on studies conducted in India [47]. Dietary diversity was measured using a version of the Food Frequency Questionnaire that asked women how often they consume a particular food item. Responses were coded on a 7-point Likert scale (0 = never, 6 = daily). There were a total of 28 food items (see electronic supplementary material, S1). Frequencies were added together to create a total score for dietary diversity.

### 5.4. Social bargaining

The social bargaining model predicts that women will crave foods that are harmful to the developing fetus. The outcome variables for this model, therefore, included both an etic and emic measure of ‘dangerous’ foods. The dichotomous toxicity/pathogenicity variable was used as the etic measure. A study by Placek & Hagen [36] found that ‘hot’ and ‘black’ foods are considered dangerous to eat during pregnancy. In this study, hot foods included sour items, unripe mango, mango, papaya, pineapple, palmyra sprouts, eggplant, chicken and fish. Black items included black grapes and jamun. A ‘hot’ and ‘black’ variable was computed from cravings to serve as the emic outcome variables. Finally, unripe mango and unripe tamarind were modelled separately because these two items were craved in high frequency.

Predictor variables included the presence or absence of pressure to have a son (yes = 1, no = 0), the total number of living children, food insecurity and psychological distress. An interaction term was added for pressure to have a son and total number of living children because these variables are often related [48]. The total score from the Kessler-6 was included as an indicator of general psychological distress. The K-6 is a six-item measure that assesses serious mental illness in World Health Organization surveys [49]. Measures tested in India have demonstrated adequate internal consistency [50] and have proven to be reliable among pregnant women residing in South India [51]. The initial item pools for the K-6 include both depression (e.g. Beck Depression Inventory) and anxiety measures (e.g. Self-Rating Anxiety Scale; [52]), and subsequent studies have used the K-6 as both a measure for depression and anxiety, but most commonly for psychological distress (e.g. [52]). Questions include symptoms of depression, fatigue, motor agitation, worthless guilt and anxiety experienced within a 30-day time frame; e.g. ‘In the last 30 days, how often did you feel worthless?’ [52]. Responses were based on a 4-point scale ranging from *none of the time* to *most of the time*.

## 6. Results

Pregnant women had an average age of 23.29 years (range: 19.00–35.00) and had completed an average of 9.20 years of school (range: 0.00–18.00). Nearly half of the women were primigravid (*n* = 44, 46.81%), whereas 44 (46.81%) had one child, four (4.2%) had two children and two (2.1%) had three children. At the time of data collection, only six participants were in their first trimester of pregnancy, 41 were in the second trimester, and 47 were in the third trimester. Table 2 presents the descriptive statistics for all study variables. According to thresholds set by Blumberg *et al*. [47], a large proportion of women were food insecure (42.6%).

**Table 2.** Descriptive statistics for study variables.

variable description	presence/yes	absence/no			
fruit cravings	81	13			
unripe mango cravings	77	17			
unripe tamarind cravings	25	69			
toxicity/pathogenicity	58	32			
black food cravings	0	94			
any nausea in pregnancy	61	33			
any vomiting in pregnancy	73	21			
pressure to have a son	23	71			

	mean	median	s.d.	min	max
calorie composite score	255.80	159.00	239.30	0.00	944.00
nutritious food cravings	0.54	0.00	0.70	0.00	3.00
hot food cravings	1.36	1.00	0.76	0.00	3.00
total score from the food insecurity scale	4.32	3.00	3.08	0.00	14.00
skinfold thickness at triceps (mm)	13.00	12.00	4.27	5.00	24.00
body mass index (kg m^−2^)	22.64	22.10	3.66	15.20	31.80
months pregnant	6.38	6.88	2.03	2.00	10.00
trimester of pregnancy	2.44	2.50	0.61	1.00	3.00
psychological distress	9.81	9.00	2.61	6.00	17.00
total number of living children	0.62	1.00	0.67	0.00	3.00

**Figure 1.**
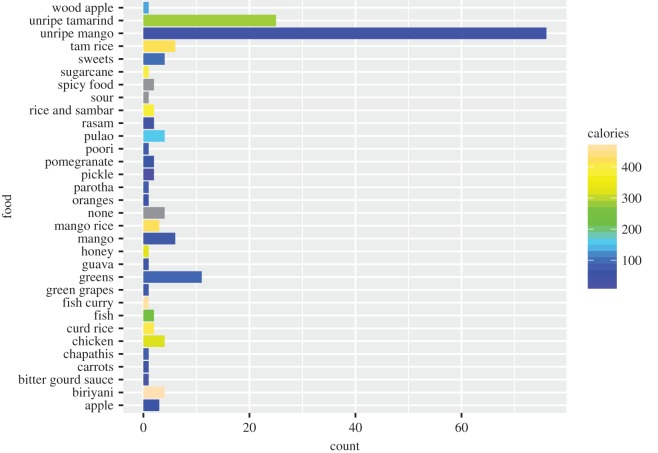
Free-listed food cravings by pregnant women and estimated calories per item.

Fifty-one women (54%) reported a craving for at least one item. The average number of cravings was 0.77 (range = 0–3). Four women (67%) in the first trimester reported at least one craving, followed by 22 women (54%) in the second trimester and 25 (61%) in the third trimester. Total cravings did not differ based on trimester (*χ*^2^ = 0.40, *p* = 0.82). The most common cravings were for unripe mango (82.0%) and unripe tamarind (26.6%). Figure 1 presents results for women's food cravings.

### 6.1. Behavioural immune system

Logistic regression was used to model the presence and absence of cravings for fruit, unripe mango and unripe tamarind. Fruit cravings were reported by 13.83% of women and were not significantly associated with the predicted measures of behavioural immunity. Unripe mango cravings were also not significantly associated with the study variables, and neither were unripe tamarind cravings. See table 3 for results, including the Nagelkerke *R*^2^ values for each model.

**Table 3.** Model results from the four study *a priori* hypotheses of specific food cravings. Coefficient estimates are presented for negative binomial and Poisson's regression models, and odds ratios are presented for logistic regression models. Model statistics include the analytical method used, along with the appropriate pseudo-*R*^2^ value. McFadden's *R*^2^ should be interpreted with caution. Significant *p*-values (*p* < 0.05) are in italics.

	estimate	odds ratio	s.e.	*Z*-value	*p* > |z|	95% CI	model statistics
**behavioural immune system**							
*model 1: fruit*							
months pregnant	0.04	1.04	0.15	0.29	0.78	0.78, 1.41	*logistic regression*
nausea	0.60	1.82	0.72	0.83	0.41	0.48, 9.01	Nagelkerke *R*^2^ = 0.02
vomiting	0.35	1.42	0.85	0.41	0.68	0.31, 10.20	
(intercept)	−2.81	0.06	1.33	−2.11	*0*.*04*	0.00, 0.70	
*model 2: unripe mango*							
months pregnant	0.09	1.10	0.14	0.68	0.50	0.84, 1.44	*logistic regression*
nausea	0.46	1.59	0.58	0.80	0.42	0.50, 4.90	Nagelkerke *R*^2^ = 0.01
vomiting	−0.45	0.64	0.73	−0.62	0.54	0.13, 2.42	
(intercept)	1.00	2.73	1.13	0.89	0.37	0.31, 27.9	
*model 3: unripe tamarind*							
months pregnant	−0.03	0.97	0.12	−0.25	0.80	0.77, 1.22	*logistic regression*
nausea	−0.03	0.97	0.51	−0.05	0.96	0.36, 2.74	Nagelkerke *R*^2^ = 0.00
vomiting	−0.14	0.87	0.58	−0.24	0.81	0.28, 2.89	
(intercept)	−0.70	0.50	0.96	−0.73	0.47	0.07, 3.22	
**nutrient seeking**							
*model 1: total calories*							
triceps thickness	0.03		0.03	0.94	0.35	0.97, 1.08	*negative binomial*
BMI	−0.03		0.03	−0.93	0.35	0.91, 1.04	McFadden's *R*^2^ = 0.06
months pregnant	0.03		0.05	0.56	0.35	0.93, 1.13	
toxicity/pathogenicity	1.06		0.18	5.92	*0*.*0001*	2.05, 4.14	
(intercept)	5.29		0.54	9.78	*0*.*0001*	70.78, 564.17	
*model 2: nutritional foods*							
triceps thickness	0.02		0.04	0.50	0.62	0.94, 1.11	*Poisson's regression*
BMI	−0.02		0.05	−0.46	0.64	0.88, 1.08	McFadden's *R*^2^ = 0.21
months pregnant	0.05		0.08	0.68	0.49	0.91, 1.22	
toxicity/pathogenicity	1.69		0.32	5.19	*0*.*0001*	2.93, 10.60	
(intercept)	−1.57		0.91	−1.73	0.08	0.03, 1.22	
**resource scarcity**							
*model 1: nutritional foods*							
triceps thickness	0.04		0.04	0.87	0.39	0.96, 1.14	*Poisson's regression*
BMI	−0.04		0.06	−0.81	0.42	0.87, 1.09	McFadden's *R*^2^ = 0.21
dietary diversity	0.00		0.01	0.27	0.79	0.99, 1.03	
food insecurity	0.06		0.05	1.08	0.28	1.02, 1.23	
months pregnant	0.09		0.09	1.03	0.30	0.88, 1.21	
toxicity/pathogenicity	1.63		0.33	4.94	*0*.*0001*	2.74, 10.13	
(intercept)	−2.06		1.32	−1.56	0.12	0.01, 1.63	
**social bargaining**							
*model 1: toxin/pathogen*							
psychological distress	−0.01	0.99	0.11	−0.07	0.95	0.79, 1.24	*logistic regression*
food insecurity	0.25	1.29	0.10	2.42	*0*.*02*	1.06, 1.60	Nagelkerke *R*^2^ = 0.25
pressure to have a son	−2.70	0.07	1.03	−2.61	*0*.*01*	0.01, 0.41	
number of living children	−1.18	0.31	0.49	−2.38	*0*.*02*	0.11, 0.77	
pressure × living children	1.58	4.75	0.81	1.92	0.06	0.98, 25.99	
(intercept)	−0.72	0.49	1.00	−0.72	0.47	0.07, 3.43	
*model 2: ‘hot’ foods*							
psychological distress	0.04		0.04	1.03	0.30	0.96, 1.13	*Poisson's regression*
food insecurity	−0.002		0.04	−0.07	0.95	0.93, 1.07	McFadden's *R*^2^ = 0.01
pressure to have a son	−0.29		0.31	−0.94	0.35	0.40, 1.34	
number of living children	−0.19		0.18	−1.08	0.28	0.57, 1.16	
pressure × living children	0.30		0.29	1.05	0.29	0.77, 2.36	
(Intercept)	0.02		0.37	0.06	0.95	0.50, 2.10	
*model 3: unripe mango*							
psychological distress	0.19	1.21	0.14	1.37	0.17	0.93, 1.63	*logistic regression*
food insecurity	0.03	1.03	0.12	0.22	0.83	0.81, 1.34	Nagelkerke *R*^2^ = 0.07
pressure to have a son	−0.48	0.62	0.87	−0.55	0.58	0.12, 3.86	
number of living children	0.45	1.56	0.61	0.73	0.47	0.50, 5.73	
pressure × living children	−0.25	0.78	0.91	−0.28	0.78	0.13, 4.95	
(intercept)	−0.50	0.61	1.20	−0.41	0.68	0.05, 6.15	
*model 4: unripe tamarind*							
psychological distress	0.16	1.17	0.12	1.36	0.17	0.93, 1.48	*logistic regression*
food insecurity	−0.10	0.91	0.10	−0.89	0.37	0.74, 1.11	Nagelkerke *R*^2^ = 0.11
pressure to have a son	−0.37	0.69	0.78	−0.47	0.64	0.13, 3.04	
number of living children	−1.19	0.31	0.59	−2.10	*0*.*04*	0.09, 0.90	
pressure × living children	1.27	3.57	0.84	1.51	0.13	0.68, 19.49	
(intercept)	−1.73	0.18	1.01	−1.71	0.09	0.02, 1.25	

### 6.2. Nutrient seeking

To test the nutrient seeking hypothesis, each food item was ranked according to calories per serving (see electronic supplementary material, S2). These results are also presented in figure 1 and show that unripe mango, the most frequently craved item, has an average of 100 calories per serving. Although unripe tamarind, the second most frequently craved item, has higher caloric density, the items with the highest calories (e.g. fish curry and biriyani) were mentioned less frequently. Results from the negative binomial regression revealed that the total score for calories of craved foods was not significantly predicted by months pregnant, triceps thickness or BMI. Interestingly, the presence of toxins or pathogens in each craved item was positively and significantly associated with the total calories composite score, indicating that cravings for calorie-rich items were confounded by possible toxicity and/or pathogenicity (Est. = 1.06, *p* < 3.09 × 10^−9^, 95% CI = 2.05, 4.14). McFadden's *R*^2^ indicated that little variance was explained by this model (*R*^2^ = 0.060).

Poisson's regression was used to model cravings for nutritious foods. Nutritional food cravings were not significantly associated with months pregnant, triceps thickness, or BMI; however, the presence of toxins or pathogens was significant (Est. = 1.69, *p* < 2.08 × 10^−7^, 95% CI = 2.93, 10.6). This model predicted 20.7% variance in cravings for nutritional foods based on McFadden's *R*^2^ (table 3).

### 6.3. Resource scarcity

Poisson regression was used to test the resource scarcity hypothesis. Results indicated that none of the main effects were significant: food insecurity, dietary diversity, triceps or BMI. As predicted, months pregnant was also not significant. Toxicity/pathogenicity was a significant confound (Est. = 1.63, *p* < 7.7 × 10^−7^, 95% CI = 2.74, 10.13). The McFadden's *R*^2^ value was 0.21.

### 6.4. Social bargaining

**Figure 2.**
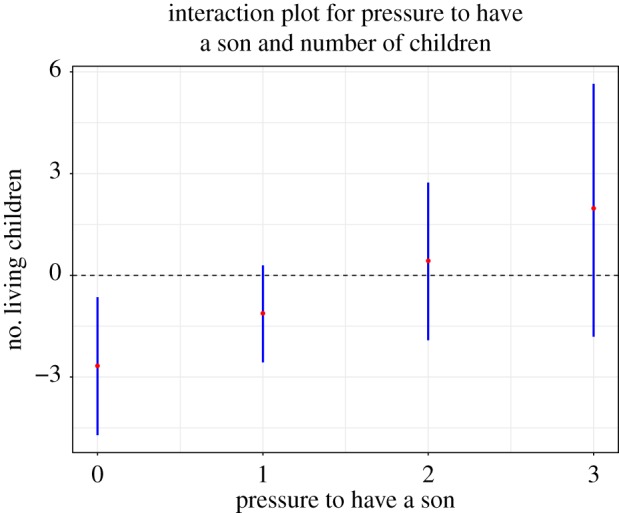
Interaction for pressure to have a son and number of children in predicting cravings for toxic and/or pathogenic food items. The graph includes 95% CIs.

Thirty-four per cent of women reported craving at least one pathogenic or toxic food item, 0% reported cravings for ‘black’ foods and 90% of women reported a craving for at least one ‘hot’ item. Logistic regression was used to analyse etic toxic/pathogenic food cravings. As predicted, food insecurity was positively and significantly associated with cravings for dangerous foods (OR = 1.29, *p* < 0.02; 95% CI = 1.06, 1.6), and the main effects for pressure to have a son and number of children predicted lower odds of craving potentially toxic or pathogenic foods (OR = 0.07, *p* < 0.01; 95% CI = 0.01, 0.41; OR = 0.31, *p* < 0.02; 95% CI = 0.11, 0.77, respectively). Psychological distress was not a significant indicator. The interaction of pressure to have a son and number of living children was marginally significant (OR = 4.75, *p* < 0.06; 95% CI = 0.98, 25.99). Figure 2 displays the interaction effect of pressure to have a son and number of children, and as predicted, as the number of children increases, the pressure to have a son also increases. The Nagelkerke *R*^2^ was 0.25 (table 3).

Poisson's regression was used to model cravings for ‘hot’ food items. None of the variables were significant. McFadden's *R*^2^ value was low at 0.01. Unripe mangoes were also not significantly predicted by any of the study variables. This model only predicted 0.07% of the variance in unripe mango consumption according to the Nagelkerke *R*^2^.

Number of living children predicted greater odds of consuming unripe tamarind (OR = 0.31, *p* < 0.04; 95% CI = 0.09, 0.9). The remaining predictors were not significant: psychological distress, food insecurity, pressure to have a son, and the interaction effect of pressure to have a son and number of living children. The Nagelkerke *R*^2^ for unripe tamarind consumption was 0.11.

Table 3 presents the results for all study models.

## 7. Discussion

This study investigated four hypotheses for food cravings in pregnancy: behavioural immunity, nutrient seeking, resource scarcity and bargaining. Similar to existing studies on pregnancy cravings, women were most likely to experience cravings for fruits [2,18,21]. Interestingly, cravings focused on unripe mango and unripe tamarind, two items that are high in antioxidants [53,54], yet culturally perceived as dangerous for the fetus if consumed in large quantities [13]. Although these cravings seem reflective of a behavioural immune strategy, the remaining analyses did not support this hypothesis, perhaps because few women were in their first trimester of pregnancy, or more importantly—these cravings likely serve an alternative purpose. The bargaining model also failed to provide strong support for unripe fruit cravings.

Cravings for unripe fruits in pregnancy are common across several states in India [55]. In Karnataka, cravings for unripe mango are locally perceived to reflect a need to reduce bodily heat, or *pitta* according to Ayurvedic theory [56], whereas in Tamil Nadu, cravings for unripe tamarinds are considered to be heat-causing and, therefore, harmful to consume in pregnancy [57]. The sour taste of fruits like these are sometimes appealing to pregnant women due to gustatory changes [58] and could signal an increased need for vitamin C [59]. Fruits rich in vitamin C aid in the absorption of iron, and iron requirements increase during pregnancy due to the maternal–fetal transfer of iron and changes in haemodilution [60]. Iron deficiency anaemia is a major public health concern for Indian women of reproductive age and higher among pregnant women who have had multiple pregnancies [61]. These fruits are rich in antioxidants, help stabilize red blood cells [53,54,62], and have highest vitamin C content during the unripe state [63]. Therefore, perhaps these cravings enable women to increase iron bioavailability, particularly among those who have higher fertility, as this study found for unripe tamarind cravings.

The social bargaining model predicted the etic classification of potentially toxic and/or pathogenic items, suggesting that women crave these foods in pregnancy when social and material resources are unstable. Psychological distress was not a significant predictor, however, which is surprising given the evidence that depressed mood states commonly reflect bargaining strategies [64,65], particularly among individuals who lack the physicality for aggressive manipulation [66]. Furthermore, this study found that the presence of toxins and/or pathogens in foods was a confounding factor for calorie-dense food and nutritional food cravings. This finding raises questions about the validity of the nutrient seeking and resource scarcity hypotheses because instead of seeking nutritious foods to protect the pregnancy, women appear to be seeking potentially teratogenic foods when resources are inadequate.

This study was an observational design and, therefore, cannot assume causality. Furthermore, only a small proportion of the participants were in their first trimester of pregnancy, which limits the ability to sufficiently test the behavioural immune hypothesis. Per the social bargaining model, more research is needed to see if the food cravings are successful in eliciting investment. The inclusion of a more precise measure of food consumption to see how nutrition influences pregnancy desires would also enhance this study. Finally, these findings are specific to pregnant women in Tamil Nadu and might not generalize to other populations of pregnant women in India and other locations. Regardless of these limitations, this study contributes to a growing literature on South Indian women that investigates how current environmental and social factors influence hypothesized dietary adaptations in pregnancy. These studies are revealing that dietary habits do not serve a single evolutionary function to prevent the ingestion of toxins and pathogens, but nonetheless, probably aim to protect the mother and/or developing fetus [13,36,67].

To conclude, although pregnancy cravings are a common phenomenon that occurs across populations, our understanding of the causes remains limited [1]. Cravings are likely to vary according to resource availability, psychosocial distress, pathogen exposure, familial support and nutritional need. This study, for instance, demonstrated that pregnancy cravings for toxic and pathogenic items most likely reflect a bargaining strategy among women who face increased social pressure to conform to cultural standards of the ‘ideal’ family. Another study conducted among these women in South India found that cravings for soils and other pica substances were a response to psychological distress and resource scarcity [13]. Further, women in the United States experience cravings for calorie-rich items, which may or may not be due to resource scarcity, but has implications for gestational weight gain that is difficult to lose during the postpartum period [1]. Collectively, these studies demonstrate that future research should consider the cultural and environmental niches that frame pregnancy cravings. Rather than focusing on biological causes of women's dietary abnormalities, followed with biomedical treatments, for instance, iron tablets to treat iron deficiency, public health efforts will be improved through systematic investigations of the evolutionary processes and cultural factors that shape consumption patterns.

## Supplementary Material

Electronic Supplementary Material 1

## Supplementary Material

Electronic Supplementary Material 2

## Data accessibility

Data are publicly available on Figshare.com (https://doi.org/10.6084/m9.figshare.4751149).
