# Supplementary material for: A test of four evolutionary hypotheses of pregnancy food cravings: evidence for the social bargaining model
Source: R Soc Open Sci. 2017 Oct 18;4(10):170243. doi: 10.1098/rsos.170243 (PMC5666241; doi:10.1098/rsos.170243)
Supplement: Electronic Supplementary Material 1 [file rsos170243supp1.docx]

Electronic Supplementary Material 1

*Summary statistics for individual Food Frequency Questionnaire items*

| **Food** | **Mean** | **Median** | **SD** | **Min** | **Max** |
| --- | --- | --- | --- | --- | --- |
| Idli/Dosa | 5.31 | 5.00 | 1.44 | 1 | 7 |
| Tea/Coffee | 4.88 | 7.00 | 2.85 | 1 | 7 |
| Sweets | 2.30 | 2.00 | 1.45 | 1 | 7 |
| Plain Rice | 6.84 | 7.00 | 0.90 | 1 | 7 |
| “Variety” Rice | 3.93 | 4.00 | 1.61 | 1 | 7 |
| Chapatti | 3.24 | 3.00 | 1.85 | 1 | 7 |
| Eggs | 4.20 | 5.00 | 1.91 | 1 | 7 |
| Groundnuts | 3.62 | 3.00 | 2.01 | 1 | 7 |
| Mango | 4.19 | 5.00 | 1.77 | 1 | 7 |
| Other Fruits | 4.00 | 5.00 | 2.02 | 1 | 7 |
| Chicken | 2.66 | 2.50 | 1.72 | 1 | 7 |
| Milk/Lassi | 4.85 | 6.00 | 2.57 | 1 | 7 |
| Potatoes | 4.00 | 5.00 | 1.71 | 1 | 7 |
| Bread | 1.78 | 1.00 | 1.56 | 1 | 7 |
| Jam | 1.44 | 1.00 | 1.31 | 1 | 7 |
| Sweet Beverages | 3.01 | 3.00 | 1.91 | 1 | 7 |
| Health Drinks | 4.62 | 5.00 | 2.23 | 1 | 7 |
| Lentils and other legumes | 2.46 | 1.00 | 2.00 | 1 | 7 |
| Noodles | 1.73 | 1.00 | 1.38 | 1 | 7 |
| Boost | 2.69 | 1.00 | 2.57 | 1 | 7 |
| Curds | 3.35 | 1.50 | 2.51 | 1 | 7 |
| Rasam | 6.00 | 6.00 | 0.98 | 1 | 7 |
| Sambar | 6.21 | 7.00 | 1.19 | 1 | 7 |
| Snacks | 2.80 | 3.00 | 1.59 | 1 | 7 |
| *Kul* | 4.65 | 5.00 | 2.40 | 1 | 7 |
| Tamarind Sauce | 2.69 | 1.50 | 1.86 | 1 | 7 |
| Spicy food | 4.67 | 5.00 | 2.07 | 1 | 7 |
| *Kangee* | 3.15 | 2.50 | 2.24 | 1 | 7 |
