# Supplementary material for: A test of four evolutionary hypotheses of pregnancy food cravings: evidence for the social bargaining model
Source: R Soc Open Sci. 2017 Oct 18;4(10):170243. doi: 10.1098/rsos.170243 (PMC5666241; doi:10.1098/rsos.170243)
Supplement: Electronic Supplementary Material 2 [file rsos170243supp2.docx]

**Electronic Supplementary Material 2**

*Calories per serving of food item*

| **Food** | **Calories** |
| --- | --- |
| Apple | 59 |
| Biriyani | 470 |
| Bitter Gourd Sauce | 25 |
| Carrots | 48 |
| Chapathis | 80 |
| Chicken | 314 |
| Curd Rice | 406 |
| Fish Curry | 460 |
| Fish | 240 |
| Green Grapes | 71 |
| Greens | 92 |
| Guava | 51 |
| Honey | 319 |
| Mango | 74 |
| Mango Rice | 420 |
| None | . |
| Oranges | 48 |
| Parotha | 80 |
| Pickle | 9 |
| Pomegranate | 65 |
| Poori | 80 |
| Pulao | 160 |
| Rasam | 36 |
| Rice & Sambar | 382 |
| Sour | . |
| Spicy Food | . |
| Sugarcane | 398 |
| Sweets | 100 |
| Tam Rice | 415 |
| Unripe Mango | 44 |
| Unripe Tamarind | 283 |
| Wood Apple | 134 |
